# Overexpressed or intraperitoneally injected human transferrin prevents photoreceptor degeneration in rd10 mice

**Published:** 2010-12-08

**Authors:** Emilie Picard, Laurent Jonet, Claire Sergeant, Marie-Hélène Vesvres, Francine Behar-Cohen, Yves Courtois, Jean-Claude Jeanny

**Affiliations:** 1 Inserm, Paris, France; 2 Centre de Recherche des Cordeliers, Université Pierre et Marie Curie, Paris, France; 3 Université Paris Descartes, Paris, France; 4 Université Bordeaux/CNRS-UMR 5084, Analytical and Bioenvironmental Nuclear Chemistry, Bordeaux-Gradignan, France

## Abstract

**Purpose:**

Retinal degeneration has been associated with iron accumulation in age-related macular degeneration (AMD), and in several rodent models that had one or several iron regulating protein impairments. We investigated the iron concentration and the protective role of human transferrin (hTf) in rd10 mice, a model of retinal degeneration.

**Methods:**

The proton-induced X-ray emission (PIXE) method was used to quantify iron in rd10 mice 2, 3, and 4 weeks after birth. We generated mice with the β-phosphodiesterase mutation and hTf expression by crossbreeding rd10 mice with TghTf mice (rd10/hTf mice). The photoreceptor loss and apoptosis were evaluated by terminal deoxynucleotidyl transferase dUTP nick end labeling in 3-week-old rd10/hTf mice and compared with 3-week-old rd10 mice. The neuroprotective effect of hTf was analyzed in 5-day-old rd10 mice treated by intraperitoneal administration with hTf for up to 25 days. The retinal hTf concentrations and the thickness of the outer nuclear layer were quantified in all treated mice at 25 days postnatally.

**Results:**

PIXE analysis demonstrated an age-dependent iron accumulation in the photoreceptors of rd10 mice. The rd10/hTf mice had the rd10 mutation, expressed high levels of hTf, and showed a significant decrease in photoreceptor death. In addition, rd10 mice intraperitoneally treated with hTf resulted in the retinal presence of hTf and a dose-dependent reduction in photoreceptor degeneration.

**Conclusions:**

Our results suggest that iron accumulation in the retinas of rd10 mutant mice is associated with photoreceptor degeneration. For the first time, the enhanced survival of cones and rods in the retina of this model has been demonstrated through overexpression or systemic administration of hTf. This study highlights the therapeutic potential of Tf to inhibit iron-induced photoreceptor cell death observed in degenerative diseases such as retinitis pigmentosa and age-related macular degeneration.

## Introduction

All cells require iron for survival and as a cofactor of a variety of enzymes [1]. Ferrous iron (Fe^2+^) reacts with H_2_O_2_ in the Fenton reaction to produce the highly reactive hydroxyl radical, which can damage proteins, lipids, and nucleic acids. Iron retinal homeostasis is regulated by proteins involved in iron import (transferrin [Tf], transferrin receptor), storage (ferritin) and export (ceruloplasmin, ferroportin, hephaestin), thus preventing deleterious consequences of either iron overload or deficiency. The study of the iron metabolism in rodent retinas has been partially elucidated by the localization of iron in the different retinal layers and by the determination of the various proteins involved in its homeostasis [2,3]. Tf is mainly expressed in the retinal pigmented epithelium (RPE) and in photoreceptors (PRs). Tf receptor and ferritin are present in all outer retinal layers [2]. Ceruloplasmin, hephaestin, and hepcidin have also been identified in retinas [4,5].

Diseases such as aceruloplasminemia and age-related macular degeneration (AMD) are associated with increased intraocular iron levels, which contribute to oxidative injury and subsequent retinal degeneration [6-8]. In fact, iron is found to be increased in the RPE, Bruch's membrane, and PR layers from AMD patients [9]. In addition, maculas of the eyes from patients with geographic atrophy also have shown elevated levels of Tf, ferritin, and ferroportin in the PRs and along the internal limiting membrane [10,11].

Iron retinal accumulation is found in rodent models of retinal degeneration caused by retinal gene mutation. In Royal College of Surgeons (RCS) rats, with disruption of Mertk tyrosine kinase receptor, iron accumulation in PR segments is accompanied by Tf degradation [12]. Rd10 mice present retinal degeneration caused by mutation in exon 13 of the β-subunit of the rod phosphodiesterase (βPDE) gene [13,14]. Recently, Deleon E. et al. [15] showed increased expression of Tf, ceruloplasmin, ferritin, and Tf receptor, and increased levels of total retinal iron and ferritin-bound iron in rd10 mice.

Tf is an extracellular protein, which has a central role in iron homeostasis by binding and transferring iron within and across tissues. Moreover, Tf by its capacity to chelate iron may protect the retina from the potentially toxic effects of unbound iron. In transgenic mice (Tg) carrying the complete human Tf (*hTf*) gene (TghTf mice), *hTf* mRNA has been found in hepatocytes, oligodendrocytes, and Sertoli cells of the testis [16,17]. We previously found that in these Tg mice, hTf was produced predominantly in the RPE and Müller glial cells (MGCs), as in human retinas, and protected MGCs in primary culture against iron excess [18].

Here, we studied the iron accumulation during the course of retinal degeneration in rd10 retinas by the proton-induced X-ray emission (PIXE) technique. We crossbred rd10 mice with TghTf mice to create mice with the βPDE mutation and hTf expression (rd10/hTf mice). To analyze the potential neuroprotective effect of hTf expression in these mice, we quantified the PR loss and revealed apoptosis in 3-week-old rd10/hTf mice, as compared to 3-week-old Rd10 mice. To confirm the results found in rd10/hTf mice, we performed intraperitoneal (i.p.) injections of hTf in 5-day-old rd10 mice and during the three following weeks. We measured the hTf concentration in the retinas of the injected mice and determined the outer nuclear layer (ONL) integrity by measuring the thickness of the PR nuclei layer 20 days after birth.

## Methods

### Proton-induced X-ray emission experiments–animal specimens

Rd10 mice (a gift from Jeff Boatright Ph.D., Department of Ophthalmology, Emory University School of Medicine, Atlanta, GA) and C57Bl/6J control mice 2–4 weeks old were fed with a standard laboratory diet and tap water ad libitum and maintained on 12h:12h light-dark cycle in a temperature-controlled room at 21–23 °C. All experimental procedures were performed in accordance with the Association for Research in Vision and Ophthalmology Statement for the Use of Animals in Ophthalmic and Vision Research. Mice were sacrificed by carbon dioxide inhalation or by an overdose of sodium pentobarbital (Sanofi Santé Animale, Libourne, France).

### Sample preparation

Cryo-sections of 20 µm thickness were obtained with a cryomicrotome (Leica, Rueil-Malmaison, France). Sagittal sections close to the optic nerve were collected on aluminum holders coated previously with a Formvar film (Agar Scientific LTD, Stansted, United Kindom UK), then samples were freeze-dried overnight at −30 °C, and finally stored in a dessicator over silica gel until analysis. A precise observation of the samples was made with an optical microscope (Carl Zeiss, Le Pecq, France) to choose the best-preserved and best-located zones for analysis.

### Analysis

The samples were analyzed with a nuclear microprobe in Bordeaux-Gradignan [19]. The conditions were exactly the same as our previous studies [20]. A 2.5 MeV proton beam of 800–900 pA and 5 µm diameter was used. The scanned areas were 150×150 µm^2^. X-rays were detected using an 80 mm^2^ Si(Li) solid-state detector (Link Analytical, Gif sur Yvette, France) and backscattered protons were detected with a 20 mm^2^ Si surface barrier detector placed at 135° to the beam direction. The organic mass of the analyzed samples and their thickness were calculated from the backscattered spectra using the RUMPIN code [21]. Iron (Fe), copper (Cu), zinc (Zn), and potassium (K) concentrations were calculated from the X-ray intensities with the GUPIX software [22]. Special attention was paid to the elemental losses induced by proton beam irradiation of the tissue.

After irradiation, the sections were stained with 1% toluidine blue or 4’,6 Diamidino-2-phenyl-indole (DAPI, Sigma, Saint Quentin Fallavier, France) diluted 1:2,000, and observed with an Aristoplan microscope (Leica) equipped for fluorescence. The identification of the retinal layers in the adjacent zones allowed us to recognize them in the irradiated areas. For each layer, it was possible to get the PIXE and Rutherford backscattering spectra and then to determine the element concentrations. Data were expressed in µg/g dry weight tissue ± standard error.

### Generation of rd10/hTf mice

Rd10/hTf mice were generated from rd10 mice (βPDE^−/−^) and from transgenic mice carrying the human transferrin gene (TghTf), both with the same genetic background (C57Bl/6J). TghTf mice carry the long *hTf* gene (80 kb) comprising its long 5′- and 3′-regulatory sequences and its own promoter [23]. Briefly, rd10 mice were crossbred with TghTf mice (hTf+) to produce heterozygous rd10/hTf mice (βPDE^+/−^/hTf+). Enzyme-linked immunosorbent assay (ELISA) assay permitted screening for the presence of hTf in the blood, but not for the number of copies. Heterozygous rd10/hTf mice were then crossbred with rd10 mice; only generated βPDE^−/−^/hTf+ mice were selected. βPDE^−/−^/hTf+ mice were then crossbred together for five subsequent generations. βPDE^−/−^/hTf+ mice were named rd10/hTf. To control that the crossbreeding had not affected the process of retinal degeneration observed in rd10 mice, we also crossbred rd10 mice with wild-type (WT) C57Bl/6J mice (Janvier, Le Genest St Isle, France). Generated mice, named rd10/WT, had the *βPDE* mutation, but did not express *hTf*. To check the absence or presence of the mutated *βPDE* gene, PCR of DNA was performed from tail biopsies. DNA was extracted using a DNeasy Blood and Tissues kit column (Qiagen, Courtaboeuf, France). The primers were as follows (Jeff Boatright, Emory University, personal communication, 2008): rd10 forward: (5′-TCT CAG CCC ACA ATC TCT CT-3′); rd10 reverse: (5′-AAA CTT CCC AAA TTC CAG GT-3′).

### PCR

The corresponding PCR amplification was performed in 35 cycles by denaturation at 95 °C for 15 min; annealing at 94, 55, and 72 °C, respectively, for 30 s, 30 s, and 2 min, and elongation at 72 °C for 10 min. The product obtained was purified and digested with the enzyme HhaI (Invitrogen, Cergy Pontoise, France), whose restriction site is not included in the rd10 mutant DNA [13]. After 1 h incubation with the enzyme at 37 °C, the digested DNA was run on an agarose gel for separation of short DNA fragments. The homozygous rd10 mutation was revealed by the presence of bands having the sizes of 1,257 and 715 bp; the heterozygous mutation was revealed by the 1,257, 715, 661, and 54 bp bands; and no mutation was revealed by the 1,257, 661, or 54 bp bands.

### Human transferrin injection

Five-day-old rd10 mice were intraperitoneally injected daily for 20 days, with Ca^2+^-free physiologic buffer (buffer saline sodium [BSS]) or with human apotransferrin solution (Sigma) at concentrations of 6, 12, 24, and 48 mg/ml in BSS. During the first 7 days, mice received 0.1 ml of hTf solution. The following 7 days, they received 0.2 ml and after, up to the date of sacrifice; the volume of injections was 0.3 ml. Mice were sacrificed at 25 or 47 days postnatally for histological analysis and hTf quantification in neural retina and immunohistochemistry studies.

### Human transferrin quantification

Mice were perfused through the left cardiac ventricle with 1× phosphate buffer saline (PBS). Five mg of neural retinas were incubated in lysis buffer (15 mM Tris, pH 7.9; 60 mM KCl; 15 mM NaCl; 2 mM EDTA; 0.4 mM phenylmethylsulphonyl fluoride [PMSF; Perbio Science, Brebiers, France]). After four freeze–thaw cycles, lysates were centrifuged at 5,000× g for 10 min, and supernatants were stored at −20 °C. hTf was quantified by an antibody-sandwich ELISA using, as previously described, two different polyclonal antibodies directed against hTf: sheep anti-hTf (Biodesign, distributed by Invitrogen) and rabbit anti-hTf (Florian Guillou, INRA, Tours-Nouzilly, France) [24]. The minimum limit for hTf detection was 0.1 ng/ml. The cross-reaction rate for hTf antibodies with mouse Tf was less than 0.05%. All standards and samples were assayed in triplicate. Results were reported in ng/ml of supernatant.

### Histology

Enucleated eyes from mice were fixed with 4% paraformaldehyde, 0.5% glutaraldehyde (LADD, Inland Europe, Conflans-sur-Lanterne, France), in PBS for 2 h. After fixation, samples were washed, dehydrated, and transferred into the infiltration solution of the Leica historesin embedding kit overnight at 4 °C. Samples were embedded in the embedding medium (Leica) and attached to a block holder after polymerization. Plastic sections (5 µm thick) were prepared on a microtome (Leica), stuck on slides coated with gelatin and stained 2 min with 1% toluidin blue solution. Sections were observed with an Aristoplan microscope (Leica) and photographed with a camera (Leica).

### Measurement of nuclear layer thicknesses

For comparison of retinal morphology from 3-week-old rd10, rd10/WT, and rd10/hTf mice, and for comparison of BSS- and hTf-injected 25-day-old rd10 mice, the outer nuclear layer (ONL) and inner nuclear layer (INL) thicknesses were measured in sagittal sections made every 200 µm on both sides of the optic nerve (at the inferior and superior poles) and across the whole retina. For each protocol, the ONL measurements were made with Visilog 6.2 (Noesis, Courtaboeuf, France) in three different sections close to the optic nerve from three to six eyes, and averaged out to a single value.

### Immunohistofluorescence analysis

Freshly enucleated eyes were fixed for 2 h with 4% paraformaldehyde in 1× PBS Gibco (Invitrogen), washed with PBS, mounted in Tissue-Tek O.C.T. (Siemens Medical, Puteaux, France) and frozen with dry ice. Frozen sections (10 µm) were cut on a microtome (Leica). Sections were incubated for 1 h with different primary antibodies diluted in PBS: rabbit polyclonal anti-mouse Tf (1:100; F. Guillou), rabbit polyclonal anti-hTf, (1:100, F. Guillou), anti-rhodopsine (1:200; Rho4D2, a gift from Dr. Robert S. Molday, University of British Colombia). Negative control sections were incubated with rabbit non-immune serum (Gibco) or without primary antibodies. After washing with PBS, sections were incubated for 1 h with secondary antibodies (goat anti-rabbit), labeled with Texas Red (Jackson Laboratories; distributed by TebuBio, Le Perray en Yvelines, France) or Alexa 488 (Molecular Probes; distributed by Invitrogen), both diluted 1:200 in PBS. Cones were directly labeled with peanut agglutinin conjugated with fluorescein isothiocyanate (Sigma). Nuclei were counterstained with DAPI. Labeled sections were observed under an epifluorescence microscope (Olympus, Rungis, France) and photographed with an Olympus camera using identical exposure parameters across samples to be compared.

### Terminal deoxynucleotidyl transferase dUTP nick end labeling

The terminal deoxynucleotidyl transferase-mediated biotinylated UTP nick end labeling (TUNEL) reaction was performed, along with DAPI staining. The protocol was adapted from manufacturer’s protocol (Roche Diagnostics, Meylan, France). Briefly, frozen retinal sections, obtained as described above, were fixed for 10 min with paraformaldehyde, washed with PBS 1×, and incubated for 2 min with 0.1% Triton X-100 in 0.1% sodium citrate on ice. Then, sections were incubated for 60 min at 37 °C with the reaction mixture (TUNEL enzyme plus TUNEL label [1/9]), and finally, nuclei were counterstained with DAPI.

### Statistical analysis

Statistical analysis was conducted using the Student’s *t* test. A p value <0.05 was considered statistically significant. Measurements of retinal nuclear layer thicknesses from various groups were compared with two-way ANOVA (ANOVA). Analysis was performed using GraphPad Prism 4 software. Each experimental condition was repeated three to four times on three to six samples each time. All results are presented as mean±standard error of the mean (SEM).

## Results

### Proton induced X-ray emission analysis of iron distribution in rd10 mouse retinas

**Figure 1:**
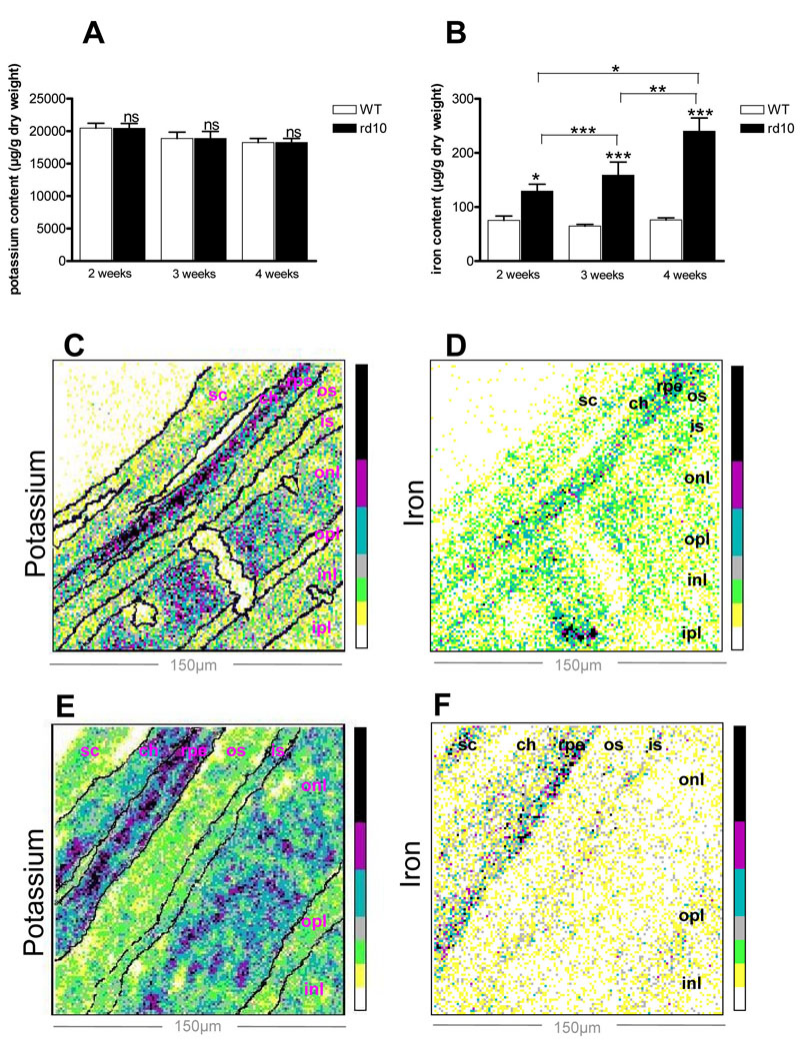
Proton-induced X-ray emission (PIXE) microanalysis of iron and potassium in 2-, 3-, and 4-week-old rd10 and wild-type mouse retinas. **A**-**B**: Concentrations in μg/g dry weight of potassium (**A**) and iron (**B**) correspond to area of photoreceptor segments from 2-, 3-, and 4-week-old rd10 and wild-type (WT) mouse retinas. White bar represents WT mice and black bar rd10 mice. Values were means±SEM (n=7–11; *p<0.05, ** p<0.01, *** p<0.001; ns: not significant). **C**-**F**: Bidimensional maps represent potassium (**C**, **E**) and iron (**D**, **F**) distribution in 4-week-old WT (**C**-**D**) and rd10 (**E**-**F**) mice. Concentrations, expressed in counts per pixel, increase according to the color scale, from white to black. The different retinal layers were identified after staining with toluidine blue and are outlined with dotted lines. Dimension of the scan are 150×150 μm^2^. Abbreviations: ch is choriocapillaris; inl is inner nuclear layer; ipl is inner plexiform layer; is is inner segments of photoreceptors; onl is outer nuclear layer; opl is outer plexiform layer; os is outer segments of photoreceptors; rpe is retinal pigmented epithelium; sc is sclera.

**Figure 2:**
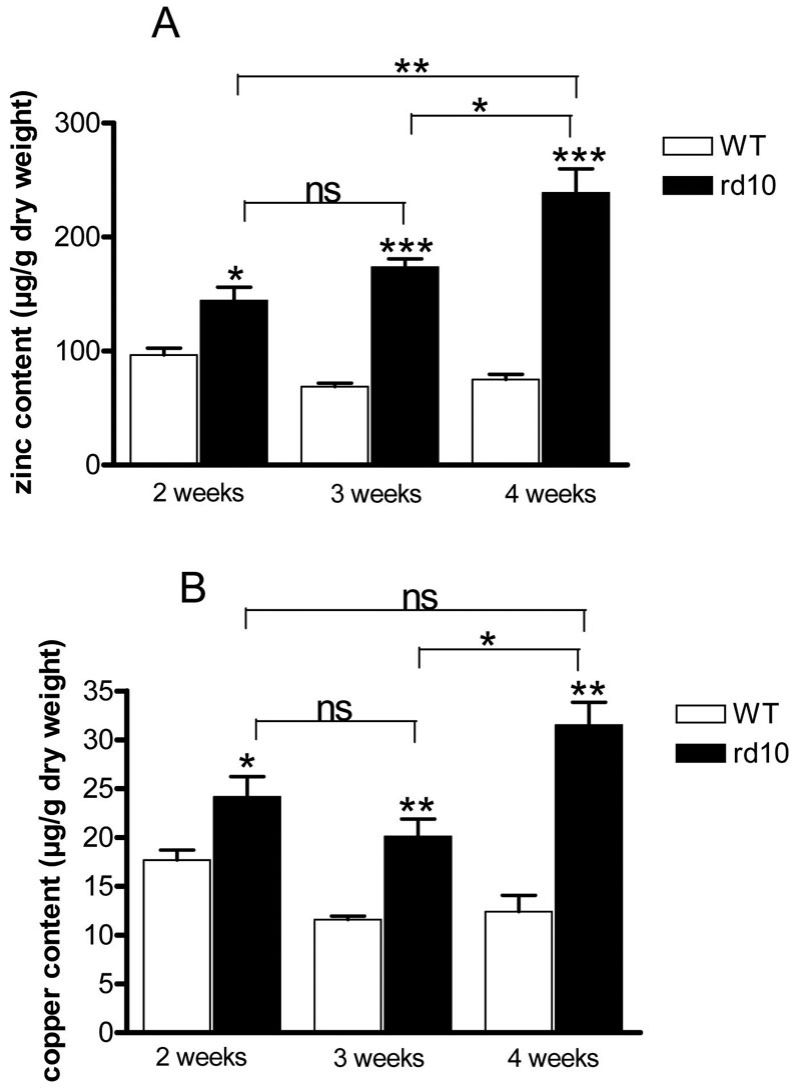
Proton-induced X-ray emission (PIXE) microanalysis of zinc and copper in 2-, 3-, and 4-week-old rd10 and wild-type mouse retinas. Concentrations of zinc (**A**) and copper (**B**) in μg/g dry weight, correspond to photoreceptor segments area from 2-, 3-, and 4-week-old rd10 and wild-type (WT) mouse retinas. The white bar represents WT mice and the black bar rd10 mice. Values were means±SEM (n=4–7; p<0.05, ** p<0.01, *** p<0.001; ns: not significant).

Iron, copper, zinc, and potassium ion contents were analyzed by PIXE microanalysis in retinas from rd10 mice and WT mice. Analysis was performed at 2, 3, and 4 weeks of age to capture early, peak, and late stages of degeneration, respectively. Potassium content was the same in both rd10 and WT mice and did not change with age between 2 and 4 weeks (Figure 1A,C,E). Total iron (heme and non-heme) content analysis demonstrated a significant increase in the area corresponding to PR inner and outer segments in rd10 mouse retinas, compared to WT mice at all ages studied. Retinal iron levels in rd10 mice compared with age-paired controls increased between 1.71, 2.45, and 3.15 fold in 2-, 3-, and 4-week-olds, respectively. Moreover, iron accumulation in the outer neural retinas of rd10 mice significantly increased in a time-dependent manner (by 23% between 2 and 3 weeks and by 51% between 3 and 4 weeks), whereas in WT mice, it remained at the same level (Figure 1B,D,F). Zinc and copper content was also significantly higher in the same area in rd10 mice compared with same-age controls. In rd10 mouse retinas, zinc increase was age-dependent, whereas copper content was statistically higher only between 3 and 4 weeks (Figure 2). Thus, PIXE analysis demonstrated that iron content, as well as zinc and copper content, was higher in rd10 mice compared to WT mice.

### Analysis of rd10/hTf mice–hTf expression

**Figure 3:**
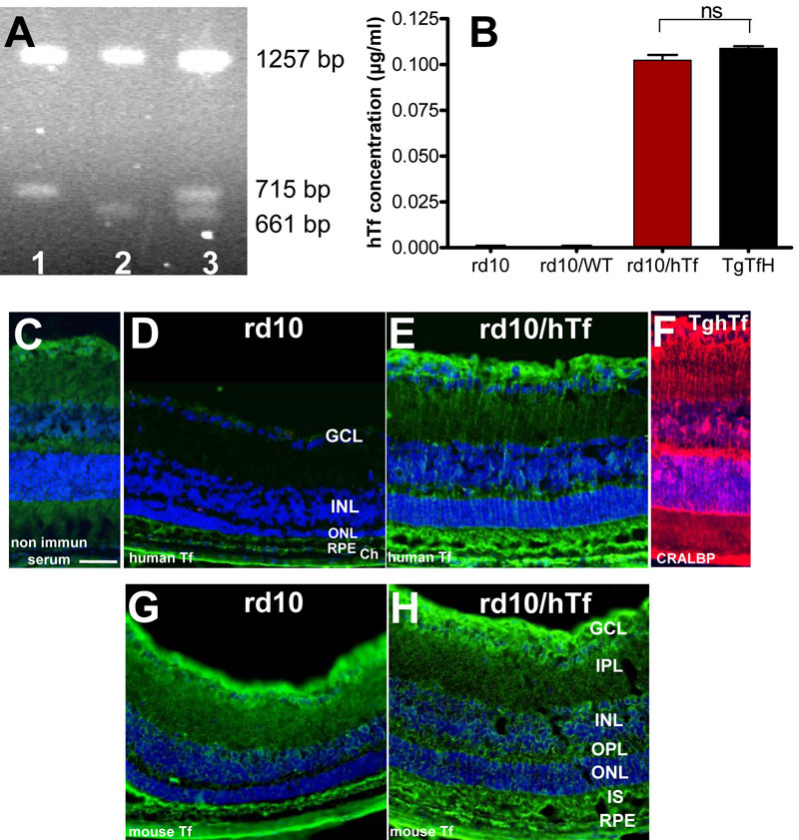
Characterization of human transferrin (hTf) expression in rd10/ hTf mouse. **A**: Gel migration of DNA extracted from products of rd10 or rd10/hTf (lane 1), TghTf (lane 2), and rd10+/−hTf (lane 3) mice use to establish the genotypes. The homozygous rd10 mutation was revealed by the presence of 1257 and 715 bp bands. The heterozygous mutation had 1257, 715, 661, and 54 bp bands. The wild-type (WT) had 1257, 661, and 54 bp bands. Bands 54 bp in size are not visible on the gel. **B**: Enzyme-linked immunosorbent assay was used to quantified hTf concentrations in lysats of neural retinas of rd10, rd10/WT, rd10/hTf, and TghTf mice. Values were means±SEM n=12; ns means not significant. **C**-**F**: hTf was immunolocalized on frozen sections of 3-week-old rd10 (**D**) and rd10/hTf (**E**) mouse retinas. Non-immune serum was used as a control (**C**). TghTf mouse retina was labeled with anti-CRALBP, a specific marker for Müller glial cell (**F**). **G**-**H**: Mouse Tf was immunolocalized on frozen sections of 3-week-old rd10 (**G**) and rd10/hTf (**H**) mouse retinas. Abbreviations: Ch is choroid; GCL is ganglion cell layer; INL is inner nuclear layer; IPL is inner plexiform layer; IS is inner segment; ONL is outer nuclear layer; OPL is outer plexiform layer; RPE is retinal pigmented epithelium. Scale bars in **C**-**H** equal 50 μm.

We generated rd10/hTf and rd10/WT mice by crossbreeding rd10 mice with TghTf mice or WT mice. As seen in Figure 3A, genotyping allows detection of the βPDE mutation. We quantified the concentration of hTf in the neural retinas of 3-week-old rd10, rd10/WT, and rd10/hTf mice, and compared the results with those from TghTf mice. As expected, there was no hTf expression in rd10 and rd10/WT mouse eyes. In neural retinas, the hTf concentration in rd10/hTf mice was similar to that in TghTf mice (Figure 3B). These results were confirmed by immunodetection of hTf in retinal sections of the four types of mice. hTf staining was not observed in retinas of rd10 mice (Figure 3D) similarly to the control incubated with no immune serum (Figure 3C) and rd10/WT mice (data not shown). hTf was localized in rd10/hTf mice (Figure 3E) in the same layers previously described in TghTf mouse retinas, that is, astrocytes, MGCs, INLs, outer and inner plexiform layers, inner segments, and the RPE [18]. Immunodetection of cellular retinaldehyde binding protein (CRALBP), a specific marker for MGCs, allowed the observation that hTf was located in MGC bodies within the inner nuclear layer and in MG cell processes that formed radial extensions through the retina (Figure 3F). Mouse Tf was detected in the same layers in rd10 and rd10/hTf mice (Figure 3G,H). The staining was slightly higher in inner segments, inner nuclear layers, and ganglion cell layers of rd10/hTf mice, compared to rd10 mice.

### Retinal degeneration

**Figure 4:**
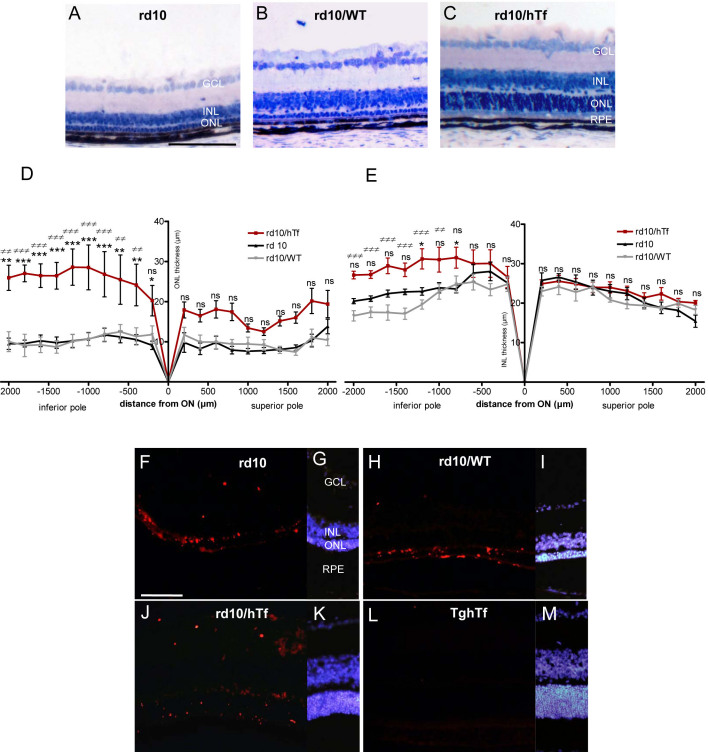
Retinal degeneration in 3-week-old rd10 mouse was compared to 3-week-old rd10/wild type (WT) and rd10/human transferrin (hTf) mice. **A**-**C**: Central part of equatorial sections represent histology from 3-week-old rd10 (**A**), rd10/WT (**B**), and rd10/hTf (**C**) mouse retinas. Nuclei were stained with toluidin blue. **D**-**E**: Outer nuclear layer (ONL; **D**) and inner nuclear layer (INL; **E**) thickness were measured every 200 μm, along the entirety of retinal sections of rd10, rd10/WT, and rd10/hTf mice at 3 weeks postnatally. (black line) rd10 mice, (red line) rd10/hTf mice, (gray line) rd10/WT mice. Values represent means±SEM, n=6–8. rd10/hTf mice were compared to rd10 mice: * p<0.05; **p<0.001; ***p<0.0001; ns represents not significant. rd10/hTf mice were compared to rd10/WT mice (≠≠ p<0.001; ≠≠≠ p<0.0001; ns: not significant; ON is optic nerve). **F**-**M**: Terminal deoxynucleotidyl transferase dUTP nick end labeling (TUNEL) was used to stain retinas from rd10 (**F**), rd10/WT (**H**), rd10/hTf (**J**), and TghTf (**L**) mice at 3 weeks postnatally. Nuclei were stained with 4’,6 Diamidino-2-phenyl-indole (DAPI): rd10 (**G**), rd10 WT (**I**), rd10/hTf (**K**), and TghTf (**M**). Abbreviations: GCL is ganglion cell layer; INL is inner nuclear layer; ONL is outer nuclear layer; RPE is retinal pigmented epithelium. Scale bars in **A**-**C** and **F**-**M** equal 100 μm.

We have considered retinal degeneration at the peak of degeneration for rd10 mice, corresponding to 3-week-old rd10, rd10/WT, and rd10/hTf mice (Figure 4). We analyzed PR loss and inner retina modifications by measuring ONL and INL thickness, respectively. As expected, no differences in ONL thickness were found between rd10 and rd10/WT mice (Figure 4A,B,D). However, the ONL thickness was significantly preserved in rd10/hTf mice compared to rd10 or rd10/WT mice (Figure 4C,D). This difference was only statistically significant in the inferior pole. From 400 µm to 2000 µm in the inferior pole, ONL thickness in rd10/hTf mice was 2.6-fold higher than in the two controls, rd10 and rd10/WT mice (Figure 4D). Average ONL thickness in the superior and inferior poles in 3-week-old TghTf mice was 43.9 µm (data not shown; SEM±0.56). It was not different in C57Bl/6J wild-type mice. In rd10/hTf mice, average ONL thicknesses in the superior and inferior poles were 59% and 36% lower (p<0.001 for the superior pole and p<0.05 for the inferior pole, respectively), in comparison with TghTf mice (data not shown).

In the INL at the superior pole, there was no significant difference between rd10, rd10/WT, and rd10/hTf mice. However, INL thickness at the inferior pole for rd10/hTf was 1.3 and 1.5 times higher than in rd10 mice and rd10/WT mice, respectively (Figure 4E). No significant differences were found between the INL thicknesses in the two poles in rd10/hTf and TghTf mice (data not shown).

We also detected apoptosis in PR nuclei by the TUNEL method in 3-week-old rd10, rd10/WT, rd10/hTf, and TghTf mice (Figure 4F-M). In the medial portion of the inferior pole, many PR nuclei in apoptosis in rd10 and rd10/WT mice were observed (Figure 4F,H), whereas only a few TUNEL-positive PR nuclei were detected in rd10/hTf mice, and none were detected in TghTf mouse retinas (Figure 4J,L).

**Figure 5:**
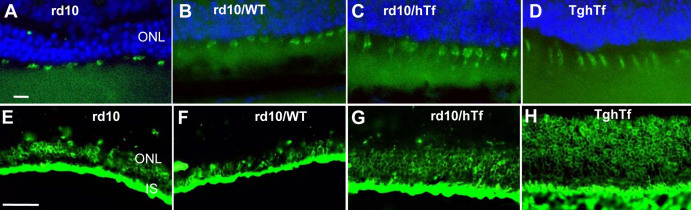
Rods and cones immunolabeling in 3-week-old rd10, rd10/wild-type (WT), rd10/human transferrin (hTf), and TghTf mice. Cones (**A**-D) and rods (**E**-**H**) were localized on frozen sections from 3-week-old rd10 (**A**, **E**), rd10/WT (**B**, **F**), rd10/hTf (**C**, **G**), and TghTf (**D**, **H**) mouse retinas. Cones were labeled with peanut-agglutinin coupled with fluorescein isothiocyanate and rods with an antibody against opsin (Rho4D2). Abbreviations: IS is inner segment; ONL is outer nuclear layer. Scale bars in **A**-**D** equal 10 μm; **E**-**H** scale bars equal 50 μm.

The distribution and the morphology of cones and rods were analyzed by immunohistofluorescence in retinal cross-sections of all types of studied mice. Rd10 and rd10/WT mouse cones labeled with peanut agglutinin seemed to be sparse, globular with collapsed outer segments (OSs), thin, small, and stunted (Figure 5A,B). However, the cones from rd10/hTf mice were shown to be more elongated and numerous than in rd10 mice (Figure 5C) and similar to those of TghTf mice (Figure 5D). On the other hand, rods immunodetected with Rho4D2 antibodies showed a similar pattern in both rd10 and rd10/WT mice (Figure 5E,F). The thickness of rod OSs was more preserved in rd10/hTf mice than in the two rd10 mice (Figure 5G). hTf expression in rd10/hTf mice seemed to protect the two types of PRs, but rods were especially protected.

### Human transferrin injection of rd10 mice–hTf retinal content

**Figure 6:**
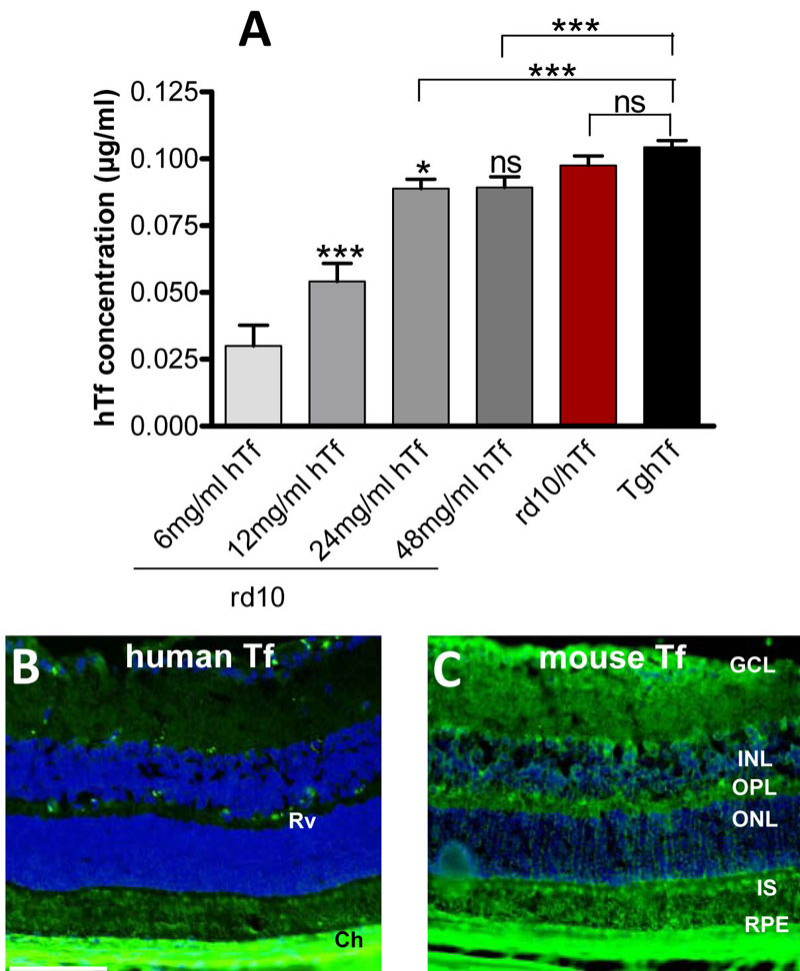
Retinal human transferrin (hTf) in rd10 mice intraperitoneally injected with hTf. **A**: Enzyme-linked immunosorbent assay (ELISA) was used to quantify hTf in lysat of neural retinas from rd10 mice daily injected intraperitoneally with 6, 12, 24, and 48 mg/ml of hTf solution from day 5 postnatally and sacrificed at 25 day postnatally. hTf concentration in rd10 mice injected was compared to hTf concentration in 3-week-old rd10/hTf and TghTf mouse retinas. Values were means±SEM, n=10. Mice injected with 12 mg/ml were compared with mice injected with 6 mg/ml. Mice injected with 24 mg/ml were compared with mice injected with 12 mg/ml. Mice injected with 48 mg/ml were compared with mice injected with 24 mg/ml. rd10/hTf mice were compared with TgTfH mice (*p<0.05; ** p<0.01; *** p<0.001; ns: not significant). **B**: hTf was immunolocalized on retinal sections of 3-week-old rd10 mice injected with 48 mg/ml of hTf. **C**: mouse Tf was immunolocalized on retinal sections of 3-week-old rd10 mice injected with 48 mg/ml of hTf. Abbreviations: Ch is choroid; GCL is ganglion cell layer; INL is inner nuclear layer; IS is inner segment; ONL is outer nuclear layer; OPL is outer plexiform layer; RPE is retinal pigmented epithelium; Rv is retinal vessel. Scale bars in **B**-**C** equal 100 μm.

We tested the potential protective role of hTf on retinal degeneration, by daily intraperitoneal injection into 5-day-old rd10 mice of four different concentrations (6, 12, 24, and 48 mg/ml) of hTf. We determined whether, after i.p. injection, hTf could cross the blood-retinal barrier and reside within the retina. The concentration of hTf was quantified by ELISA in neural retinal lysates 20 days after the beginning of daily injections, which corresponded roughly to the concentration 3 weeks postnatally and to the peak of major retinal degeneration in rd10 mice. A dose-dependent effect between the quantity of injected hTf and the local hTf concentration was found in the retina, with a plateau reached after injecting with 24 mg/ml (Figure 6A). In rd10 mice injected with 48 mg/ml of hTf, hTf staining was observed in retinal vessels and choriocapillaries (compare Figure 6B with Figure 3D). Mouse Tf was present throughout the retina and strongly localized in the ganglion cell layer, at the inner top of the inner nuclear layer, in the outer plexiform layer, and in the inner segment (Figure 6C).

### Retinal degeneration

**Figure 7:**
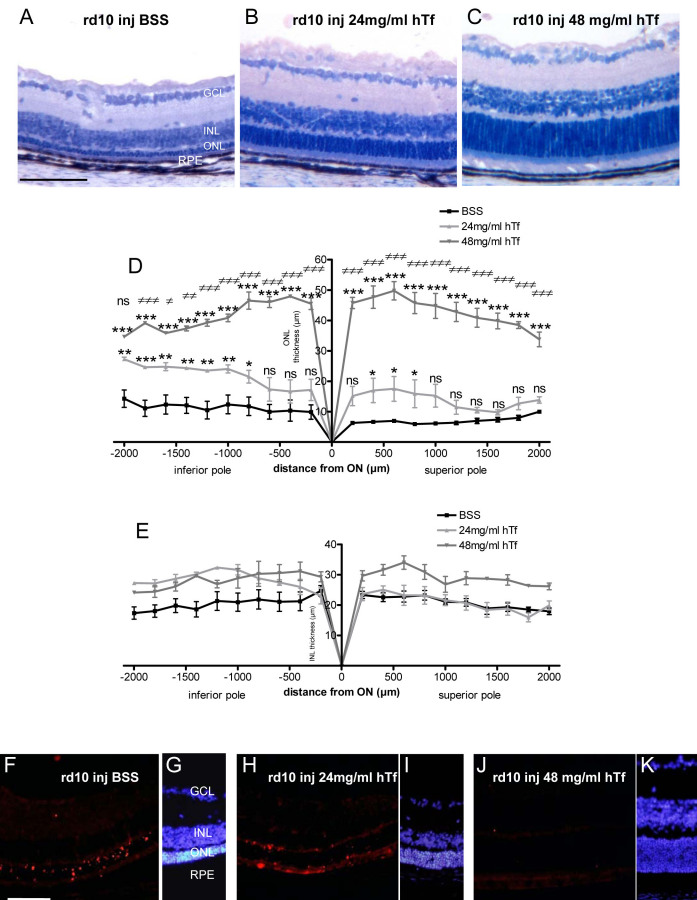
Retinal degeneration in 3-week-old rd10 mice injected with buffer saline sodium (BSS) or human transferrin (hTf). **A**-**C**: Retinal central sections represent histology of 25-day-old rd10 mice injected with BSS (**A**) or 24 mg/ml (**B**) and 48 mg/ml of hTf (**C**). Nuclei were stained with toluidin blue. **D**-**E**: Outer nuclear layer (ONL; **D**) and inner nuclear layer (INL; **E**) thickness were measured every 200 μm, along the whole retinal section from 5-day-old rd10 mice daily injected intraperitoneally with BSS and 24 and 48 mg/ml of hTf, and sacrificed at 25 days postnatally. (black line) rd10 mice injected with BSS, (light gray line) rd10 mice injected with 24 mg/ml of hTf, (dark gray line) rd10 mice injected with 48 mg/ml of hTf. Values represent means±SEM, n=5–8. **D**: rd10 mice injected with 24 mg/ml of hTf were compared to mice injected with BSS: * p<0.05; **p<0.001; ***p<0.0001; ns represent not significant. rd10 mice injected with 48 mg/ml of hTf were compared to mice injected with BSS: ***p<0.0001. rd10 mice injected with 48 mg/ml of hTf were compared to mice injected with 24 mg/ml of hTf: ≠ p<0.05; ≠≠ p<0.001; ≠≠≠ p<0.0001; ns: not significant. **E**: The differences are mainly insignificant (ON: optic nerve). **F**-**K**: Terminal deoxynucleotidyl transferase dUTP nick end labeling (TUNEL) was used to stain retina from mice intraperitoneally injected every day since day 5 with BSS (**F**), or with 24 mg/ml of hTf (**H**) and 48 mg/ml (**J**) of hTf, and sacrificed at 25 days postnatally. Nuclei were stained with 4’,6 Diamidino-2-phenyl-indole (DAPI) on retinal sections from rd10 mice injected with BSS (**G**), 24 (**I**) and 48 mg/ml (**K**) of hTf. Abbreviations: GCL is ganglion cell layer; INL is inner nuclear layer; ONL is outer nuclear layer; RPE is retinal pigmented epithelium. Scale bars in **A**-**C** and **F**-**K** equal=100 μm.

The neuroprotective capacity of hTf was determined by measuring the ONL and the INL thickness every 200 μm on the totality of superior and inferior poles in the retinas of rd10 mice injected with vehicle (BSS) or hTf solutions at 24 and 48 mg/ml. As we observed in the retinal sections from 25-day-old rd10 mice treated with BSS and stained with toluidin blue, the ONL completely disappeared, and only one to two rows of PR nuclei remained close to the RPE layer (Figure 7A). The treatment with 24 mg/ml and in major degree with 48 mg/ml of hTf rescued the overall structure, demonstrated by the length of the OS as well as the density of rod nuclei (or the ONL thickness; Figure 7B-C). A precise analysis of nuclear layer thickness in animals treated with 24 mg/ml of hTf illustrated that the ONL was preserved in the major part of the retina. However, it was preserved statistically more significantly both between 400 and 800 µm at the superior pole (on average, it was 2.5 fold higher in mice injected with 24 mg/ml hTf than those injected with BSS) and between 800 and 2,000 µm at the inferior pole (on average, twofold higher in mice injected with 24 mg/ml hTf than those injected with BSS; Figure 7D). In rd10 mice injected with 48 mg/ml of hTf, the ONL thickness was significantly higher, between 6.3 and 3.7 fold, at the superior and inferior poles, respectively, than in rd10 mice injected with BSS (Figure 7D). The ONL thickness of rd10 mice injected with 48 mg/ml of hTf was not statistically different from the ONL in the two poles in TghTf mice (data not shown).

Analysis of the INL revealed that the superior pole shows the same pattern of retinal thickness in rd10 mice injected with BSS or with 24 mg/ml of hTf, but it was much higher in the inferior pole. By contrast, the INL thickness of rd10 mice injected with 48 mg/ml of hTf showed a pattern thicker in both poles than did that of mice injected with BSS (Figure 7E). There was no difference between the mean INL thickness of rd10 mice injected with 48 mg/ml hTf and TghTf mice (data not shown).

These results were confirmed by PR apoptosis detection in retinal sections of rd10 mice injected with BSS or with hTf, and evaluated by TUNEL reaction. We detected many PR nuclei in apoptosis in the ONL of rd10 mice injected with BSS, whereas in hTf injected rd10 mice, only a few apoptotic (TUNEL positive) nuclei were found (Figure 7F-K). Injection of hTf had a protective effect on retinal degeneration, by reducing the loss of PRs by apoptosis.

**Figure 8:**
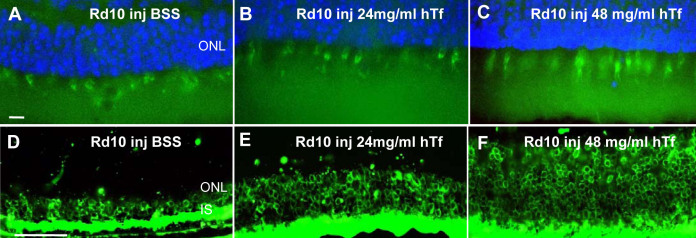
Rods and cones distribution in 3-week-old rd10 mice injected with buffer saline sodium or human transferrin. Cones (**A**-**C**) and rods (**D**-**F**) were localized on frozen sections from mice daily intraperitoneally injected with BSS (**A**, **D**) or 24 mg/ml (**B**, **E**), and 48 mg/ml (**C**, **F**) of hTf since day 5 and sacrificed at 25 days postnatally. Cones were labeled with peanut-agglutinin coupled with fluorescein isothiocyanate (FITC), and rods with antibodies against rhodopsin (Rho4D2). Abbreviations: IS is inner segment of the photoreceptors; ONL is outer nuclear layer. Scale bars in **A**-**C** equal 10 μm; in **D**-**F** they equal 50 μm.

Finally, we determined the hTf effects on PR cell rescue in rd10 mutant mice injected with BSS or with two different hTf doses (24 and 48 mg/ml). We observed that cones from rd10 mice injected with BSS were round and disorganized, similar to the non-injected rd10 mice (compare Figure 8A with Figure 5A,B). However, in rd10 animals injected with hTf, as in rd10/hTf or TghTf mice, the cones were elongated and more numerous than in rd10 mice injected with BSS (compare Figure 8B,C with Figure 5C,D). On the other hand, rods in rd10 mice injected with BSS also showed pathological characteristics of rod degeneration similar to the rods from rd10 or rd10/WT animals (compare Figure 8D with Figure 5E,F). However, in those rd10 mice injected with hTf, the number and the length of rods were clearly higher than in those mice injected with BSS. The effect was more pronounced in rd10 mice injected with a dose of 48 mg/ml than in mice injected with 24 mg/ml of hTf (Figure 8E,F). In rd10 mice treated with 24 mg/ml of hTf, labeling of rods was similar to that of rods in rd10/hTf mouse retinas (compare Figure 8E with Figure 5G). The rod layer from rd10 mice injected with 48 mg/ml of hTf was characteristically similar to that of TghTf mice (compare Figure 8F with Figure 5H). Thus, high doses of hTf showed a strong protective effect on rods’ and cones’ outer segments’ integrity.

## Discussion

Iron is an essential component of cell survival [1]; however, its capacity to generate highly reactive hydroxyl free radicals via the Fenton reaction can result in toxicity for the cells. Iron overload is found in human retinas during aging [6,25] and has been associated with retinal degeneration in AMD patients [9]. Moreover, iron homeostasis defects in aceruloplasminemia patients [26] or in Cp^−/−^Heph-/Y mice [4] are directly associated with retinal degeneration. Two rodent models of retinal degeneration caused by an inherited mutation have been demonstrated to present an iron retinal excess and a modification of iron-regulating protein expression in RCS rats with a mutation in the Mertk gene and in rd10 mice with a mutation in the βPDE gene [12,15]. The rd10 mutant is a phenotype characterized by slow retinal degeneration; it is a potential model for studying the cell biology of this disease and for testing therapeutic tools.

Results from the present study using PIXE analyses showed an alteration in iron metabolism during retinal degeneration in rd10 mice. These results were similar to those previously reported in animals evaluated when 3 weeks old [15]. We detected an increase in total (heme and non-heme) iron content in inner and outer segments of PRs from rd10 mice, compared to control mice, and its accumulation was age-dependent. The major part of total iron content may be bound in ferritin complexes, since Deleon E. et al. [15] found increased ferritin-bound iron in rd10 mouse retinas. In addition, our results showed that more than two-thirds of iron content was localized in the inner segments of PRs; these contain the metabolic machinery of PRs and mitochondria, and need iron to be available. Zinc levels were also age-dependent in the rd10 animals, whereas copper content only increased between 3 and 4 weeks postpartum. Zinc, like iron, is found in sub-RPE deposit formations in AMD patients, and it is abundant in RCS rats [9,27,28]. Human copper metabolism disorders such as Menkes disease and Wilson disease can result in retinal degeneration [29].

Our results show that degenerative processes due to genetic mutations correlate with the increased release of (bound or unbound) iron after PR death [1,3]. The excessive iron level in rd10 mice, as well as in human AMD cases, promotes itself PR death. An altered iron, zinc, and copper metabolism may play an important role in oxidative stress associated with the progression of retinal degeneration.

Furthermore, we investigated the potential protective effect of hTf in the rd10 model of retinal degeneration. We previously demonstrated, in vitro, that hTf expressed by MGCs from TghTf mice in culture or hTf added to the culture medium of MGCs from WT mice, had the same protective role against oxidative stress induced by iron excess [18]. Our study, based on these two in vitro methods, was applied in vivo to rd10 mice: one by generating rd10 mice expressing hTf and other one by injecting hTf directly into rd10 mice.

For the first strategy, we generated a mouse homozygous for the βPDE mutation and the hTf gene: the rd10/hTf mouse. This mouse expressed the same hTf content in retinas as that in TghTf mice. Moreover, the crossbreeding did not modify the hTf transgenic construct, because the protein was localized, as it is in human retinas and in TghTf mouse retinas. hTf was expressed in the whole retina, especially in the RPE and MGCs [18]. The ONL thickness was more preserved and TUNEL positive cells were less numerous in rd10/hTf mice than in rd10 mice. In addition, hTf partly preserved the number of rods and the morphology of cones. Thus, hTf expression in rd10/hTf, at the level of TghTf mouse retinas, rescued PRs being lost due to the βPDE mutation. PR death induced structural changes in neuronal postsynaptic cells, which resulted in decreased INL thickness [30]. In rd10/hTf mice, there was a higher INL thickness than in rd10 mice, demonstrating the hTf neuroprotective effect not only on PRs but also on the preservation of other neurons. The percentage of hTf protection was higher in the inferior pole than in the superior pole, which suggested a gradient-like hTf expression in rd10/hTf mouse retinas.

For the second strategy, every day we injected 24 and 48 mg/ml of apo-hTf (iron-free hTf) into 5-day-old rd10 mice before the onset of retinal degeneration. We chose the i.p. route instead of the intravitreal one to avoid the risks of ocular infection and variations in intraocular pressure. We noted that the immunostaining of hTf in mouse retinas injected with hTf (Figure 5B) was stronger in choroidal and retinal capillaries. Hunt and Davis [31] demonstrated that Tf from choriocapillaries can be bound by the RPE on the basal membrane and released to other side. Human transferrin has 50% homologous to mouse transferrin proteins, so we can suppose that hTf can be bound by mouse transferrin receptors. Burdo J.R. et al. [32] demonstrated that the route of iron delivery throughout the inner blood-retina barrier could involve Tf receptor-mediated transcytosis. The protective effect and hTf concentration in retinas were proportional to the concentration of hTf injected. The effect of hTf injection was measured 20 days after the beginning of the protocol, at 25-day-old, the peak of retinal degeneration in rd10 mice [30]. When 24 and 48 mg/ml were injected, the hTf concentration in retinas was nearly the same as that in rd10/hTf and TghTf mice. However, the retinas of rd10 mice injected with 48 mg/ml of hTf had kept almost all their PRs, compared to rd10 mice injected with BSS. The ONL thickness was nearly the same as that in TghTf and WT mice. The retinas of these animals showed only few TUNEL-positive PRs, and the cone and rod morphology was intact. Moreover, the INL thickness was similar at the INL in TghTf mice, demonstrating the preservation of secondary neurons. But in rd10 mice injected with 24 mg/ml, the PR rescue was less substantial than in 48 mg/ml hTf-injected mice. Nevertheless, the rescue of PRs was significant, compared to that in mice injected with BSS. The discrepancy in the ONL thickness rescue and the hTf concentration measured in retinas of rd10 mice injected with 24 and 48 mg/ml, could be explained by a limited quantity of Tf that could be present in the retina. The rest of the hTf remained available in the blood flow for the retina. A regulatory mechanism may exist to regulate Tf entry to retinas [33].

There was a discrepancy between the almost equal levels of hTf in the retinas of rd10/hTf mice or mice injected with hTf and the different level of PR protection. This might be explained by the limited capability of retinal cells (in which are located the RPE and MGCs) to produce hTf in rd10/hTf mice while the blood concentration of hTf in injected mice was elevated, available, and could be regulated.

The two strategies permitted presence of hTf in the retinas of mice and reduced retinal degeneration. In rd10 mouse retinas, PR death produced oxidative stress and released iron [15,34]. Deleon et al. [15] demonstrated that levels of Tf, ceruloplasmin, ferritin, and Tf receptor increased in 3-week-old rd10 mouse retinas. Thus, in response to iron excess, rd10 retinas increase Tf expression to manage and transport iron. In our study, we experimentally increased Tf levels, but to a higher level than is normal for rd10, to achieve an amount sufficient both to chelate and decrease the iron released and to decrease oxidative stress. hTf has already been studied for its potential as antioxidant. In renal ischemia-reperfusion injuries, Tf lowers the circulating redox-active iron levels [35]. Tf has potent anti-apoptotic/cytoprotective effects against Fas-mediated signals in hepatocytes and lymphohematopoietic cells [36,37]. In a rabbit model of cataracts formed after cataract surgery, Tf synthesis upregulated in lens epithelia, acting as a survival and proliferative factor [38].

Here, we also observed that in rd10/hTf mice and in rd10 mice injected with hTf, mouse Tf staining increased in the retina, especially in the inner segment and ganglion cell layer, compared to the rd10 mouse retina (Figure 2H and Figure 5C). This data confirmed our previous study [18], where we demonstrated that hTf expression in TgTfH transgenic mice increased mouse Tf expression, and enhanced a possible loop of autoregulation for transferrin expression similar to that in the two mouse species studied here.

The present study shows that hTf, an endogenous iron-binding protein, preserves rods and cones, which have been demonstrated to be highly sensitive to iron excess [39]. hTf also protected secondary neurons, and thus possibly preserved neuronal post-synaptic connections. Other iron homeostasis proteins, like heavy chains of ferritin, can also participate in the control of iron excess, as we have shown recently for aging and light-induced stress to retinas of heterozygous mice with a heavy chains of ferritin (Picard E. et al., IOVS, in press). These results show that one of the major central actors in many neuronal degenerative diseases in the eye, as in the brain, is the ability of free iron to generate free radicals and be a major part of the oxidative stress mechanism. How long such a treatment needs to be pursued to protect cones in the long-term remains to be determined, but it is already known that Tf is not toxic by itself at a high dose and when administrated over a long period. Preliminary experience demonstrates the limited role of hTf in protecting retinas over 47 days postnatally (data not shown). Is this oxidative stress mechanism also involved in the protection of interneurons (amacrin, bipolar, and horizontal cells) between PN0 and PN15 from programmed cell death during the development of the mouse retina? How do neurotrophic actors, like CNTF or FGF, interact with these iron-dependent oxidative mechanisms? This is under investigation. Tf also has multiple biologic functions not related to its iron binding capacity, but rather to other properties, such as its ability to bind insulin-like growth factor binding protein 3 [40]. With the use of a biologic agent like hTf, we could override the potential use of pharmaceutical chelators, such as deferoxamine, which are toxic at high doses, or of other chelators that have been proposed to protect retinas [41]. In this study, we found a correlation of iron homeostasis imbalance with the neurodegenerative state of retinas in rd10 mice, and showed, for the first time, a protective role for hTf in an animal model accumulating iron in PRs. Iron accumulation due to retinal degeneration was previously found in RCS rats and in AMD patients. Thus, it can be proposed that iron released after cell metabolism impairment, due for instance to a deficient phagocytosis, or cell death [1,3], will participate in a common toxic mechanism that is involved in degenerative diseases. This therapeutic strategy could be applicable to other degenerative models. We proposed that in RCS rats the defective phagocytosis disturbed iron recycling and resulted in Tf degradation [12]. Preliminary experiments on these rats demonstrated the rescue of PRs after injection of hTf [42]. Moreover, hTf injection could be envisaged in AMD therapy, where increased levels of chelatable iron was observed in the RPE and Bruch’s membrane [9]. The AREDS study [43] has already demonstrated the beneficial effect of a diet enriched with antioxidant nutrients on the development of AMD.

Intraperitoneal administration is of particular interest, since it highlights the therapeutic potential of Tf for protecting iron-induced PR death in degenerative ocular diseases.

## References

[1] Ponka P Cellular iron metabolism. Kidney Int Suppl 1999 69 S2 11 10084280 10.1046/j.1523-1755.1999.055suppl.69002.x

[2] Yefimova MG Jeanny JC Guillonneau X Keller N Nguyen-Legros J Sergeant C Guillou F Courtois Y Iron, ferritin, transferrin, and transferrin receptor in the adult rat retina. Invest Ophthalmol Vis Sci 2000 41 2343 51 10892882

[3] He X Hahn P Iacovelli J Wong R King C Bhisitkul R Massaro-Giordano M Dunaief JL Iron homeostasis and toxicity in retinal degeneration. Prog Retin Eye Res 2007 26 649 73 17921041 10.1016/j.preteyeres.2007.07.004 PMC2093950

[4] Hahn P Dentchev T Qian Y Rouault T Harris ZL Dunaief JL Immunolocalization and regulation of iron handling proteins ferritin and ferroportin in the retina. Mol Vis 2004 10 598 607 15354085

[5] Gnana-Prakasam JP Martin PM Mysona BA Roon P Smith SB Ganapathy V Hepcidin expression in mouse retina and its regulation via lipopolysaccharide/Toll-like receptor-4 pathway independent of Hfe. Biochem J 2008 411 79 88 18042040 10.1042/BJ20071377 PMC3731152

[6] Hahn P Ying GS Beard J Dunaief JL Iron levels in human retina: sex difference and increase with age. Neuroreport 2006 17 1803 6 17164668 10.1097/WNR.0b013e3280107776

[7] Klomp LW Gitlin JD Expression of the ceruloplasmin gene in the human retina and brain: implications for a pathogenic model in aceruloplasminemia. Hum Mol Genet 1996 5 1989 96 8968753 10.1093/hmg/5.12.1989

[8] Morita H Ikeda S Yamamoto K Morita S Yoshida K Nomoto S Kato M Yanagisawa N Hereditary ceruloplasmin deficiency with hemosiderosis: a clinicopathological study of a Japanese family. Ann Neurol 1995 37 646 56 7755360 10.1002/ana.410370515

[9] Hahn P Milam AH Dunaief JL Maculas affected by age-related macular degeneration contain increased chelatable iron in the retinal pigment epithelium and Bruch's membrane. Arch Ophthalmol 2003 121 1099 105 12912686 10.1001/archopht.121.8.1099

[10] Chowers I Wong R Dentchev T Farkas RH Iacovelli J Gunatilaka TL Medeiros NE Presley JB Campochiaro PA Curcio CA Dunaief JL Zack DJ The iron carrier transferrin is upregulated in retinas from patients with age-related macular degeneration. Invest Ophthalmol Vis Sci 2006 47 2135 40 16639025 10.1167/iovs.05-1135

[11] Dentchev T Hahn P Dunaief JL Strong labeling for iron and the iron-handling proteins ferritin and ferroportin in the photoreceptor layer in age-related macular degeneration. Arch Ophthalmol 2005 123 1745 6 16344450 10.1001/archopht.123.12.1745

[12] Yefimova MG Jeanny JC Keller N Sergeant C Guillonneau X Beaumont C Courtois Y Impaired retinal iron homeostasis associated with defective phagocytosis in Royal College of Surgeons rats. Invest Ophthalmol Vis Sci 2002 43 537 45 11818402

[13] Chang B Hawes NL Pardue MT German AM Hurd RE Davisson MT Nusinowitz S Rengarajan K Boyd AP Sidney SS Phillips MJ Stewart RE Chaudhury R Nickerson JM Heckenlively JR Boatright JH Two mouse retinal degenerations caused by missense mutations in the beta-subunit of rod cGMP phosphodiesterase gene. Vision Res 2007 47 624 33 17267005 10.1016/j.visres.2006.11.020 PMC2562796

[14] Gargini C Terzibasi E Mazzoni F Strettoi E Retinal organization in the retinal degeneration 10 (rd10) mutant mouse: a morphological and ERG study. J Comp Neurol 2007 500 222 38 17111372 10.1002/cne.21144 PMC2590657

[15] Deleon E Lederman M Berenstein E Meir T Chevion M Chowers I Alteration in iron metabolism during retinal degeneration in rd10 mouse. Invest Ophthalmol Vis Sci 2009 50 1360 5 18997094 10.1167/iovs.08-1856

[16] Lécureuil C Saleh MC Fontaine I Baron B Zakin MM Guillou F Transgenic mice as a model to study the regulation of human transferrin expression in Sertoli cells. Hum Reprod 2004 19 1300 7 15105395 10.1093/humrep/deh297

[17] Saleh MC Espinosa de los Monteros A de Arriba Zerpa GA Fontaine I Piaud O Djordjijevic D Baroukh N Garcia Otin AL Ortiz E Lewis S Fiette L Santambrogio P Belzung C Connor JR de Vellis J Pasquini JM Zakin MM Baron B Guillou F Myelination and motor coordination are increased in transferrin transgenic mice. J Neurosci Res 2003 72 587 94 12749023 10.1002/jnr.10619

[18] Picard E Fontaine I Jonet L Guillou F Behar-Cohen F Courtois Y Jeanny JC The protective role of transferrin in Muller glial cells after iron-induced toxicity. Mol Vis 2008 14 928 41 18509548 PMC2391081

[19] Llabador Y. Applications of nuclear Microprobe in the life sciences: an efficient analytical technique for research in biology and medicine, Eds Llabador Y, Moretto P. 1998. 274 p.

[20] Sergeant C Gouget B Llabador Y Simonoff M Yefimova M Courtois Y Jeanny JC Iron in hereditary retinal degeneration: PIXE microanalysis. Preliminary results. Nucl Instrum Methods Phys Res B 1999 158 344 8

[21] Moretto P Razafindrale L Simulation of RBS spectra for quantitative mapping of in homogenous biological tissue. Nucl Instrum Methods Phys Res B 1995 104 171 5

[22] Maxwell JA Campbell JL Teesdale WJ The Guelph PIXE software package. Nucl Instrum Methods Phys Res B 1989 43 218 30

[23] Cassia R Besnard L Fiette L Espinosa de los Monteros A Ave P Py MC Huerre M de Vellis J Zakin MM Guillou F Transferrin is an early marker of hepatic differentiation, and its expression correlates with the postnatal development of oligodendrocytes in mice. J Neurosci Res 1997 50 421 32 9364327 10.1002/(SICI)1097-4547(19971101)50:3<421::AID-JNR8>3.0.CO;2-K

[24] de Arriba Zerpa GA Saleh MC Fernández PM Guillou F Espinosa de los Monteros A de Vellis J Zakin MM Baron B Alternative splicing prevents transferrin secretion during differentiation of a human oligodendrocyte cell line. J Neurosci Res 2000 61 388 95 10931525 10.1002/1097-4547(20000815)61:4<388::AID-JNR5>3.0.CO;2-Q

[25] Sergeant C, Vesvres M-H, Pravikoff MS, Devès G, Yefimova M, Jonet L, Valtink M, Courtois Y, Jeanny J. C. Trace metals in human retina during aging period. Eds John Libbey. Metal ions in biology and medicine. 2004. Vol 10. p. 491–5.

[26] Dunaief JL Richa C Franks EP Schultze RL Aleman TS Schenck JF Zimmerman EA Brooks DG Macular degeneration in a patient with aceruloplasminemia, a disease associated with retinal iron overload. Ophthalmology 2005 112 1062 5 15882908 10.1016/j.ophtha.2004.12.029

[27] Lengyel I Flinn JM Peto T Linkous DH Cano K Bird AC Lanzirotti A Frederickson CJ van Kuijk FJ High concentration of zinc in sub-retinal pigment epithelial deposits. Exp Eye Res 2007 84 772 80 17313944 10.1016/j.exer.2006.12.015

[28] Eckhert CD Elevated body zinc in rats with inherited retinal dystrophy. J Hered 1981 72 130 7276513 10.1093/oxfordjournals.jhered.a109444

[29] Waggoner DJ Bartnikas TB Gitlin JD The role of copper in neurodegenerative disease. Neurobiol Dis 1999 6 221 30 10448050 10.1006/nbdi.1999.0250

[30] Barhoum R Martinez-Navarrete G Corrochano S Germain F Fernandez-Sanchez L de la Rosa EJ de la Villa P Cuenca N Functional and structural modifications during retinal degeneration in the rd10 mouse. Neuroscience 2008 155 698 713 18639614 10.1016/j.neuroscience.2008.06.042

[31] Hunt RC Davis AA Release of iron by human retinal pigment epithelial cells. J Cell Physiol 1992 152 102 10 1618912 10.1002/jcp.1041520114

[32] Burdo JR Antonetti DA Wolpert EB Connor JR Mechanisms and regulation of transferrin and iron transport in a model blood brain barrier system. Neuroscience 2003 121 883 90 14580938 10.1016/s0306-4522(03)00590-6

[33] García-Castiñeiras S Iron, the retina and the lens: A focused review. Exp Eye Res 2010 90 664 78 20230820 10.1016/j.exer.2010.03.003 PMC2919496

[34] Komeima K Rogers BS Campochiaro PA Antioxidants slow photoreceptor cell death in mouse models of retinitis pigmentosa. J Cell Physiol 2007 213 809 15 17520694 10.1002/jcp.21152

[35] de Vries B Walter SJ von Bonsdorff L Wolfs TG van Heurn LW Parkkinen J Buurman WA Reduction of circulating redox-active iron by apotransferrin protects against renal ischemia-reperfusion injury. Transplantation 2004 77 669 75 15021827 10.1097/01.tp.0000115002.28575.e7

[36] Lesnikov V Lesnikova M Deeg HJ Pro-apoptotic and anti-apoptotic effects of transferrin and transferrin-derived glycans on hematopoietic cells and lymphocytes. Exp Hematol 2001 29 477 89 11301188 10.1016/s0301-472x(00)00687-1

[37] Lesnikov VA Lesnikova MP Shulman HM Wilson HM Hockenbery DM Kocher M Pierpaoli W Deeg HJ Prevention of Fas-mediated hepatic failure by transferrin. Lab Invest 2004 84 342 52 14704719 10.1038/labinvest.3700035

[38] Davidson MG Harned J Grimes AM Duncan G Wormstone IM McGahan MC Transferrin in after-cataract and as a survival factor for lens epithelium. Exp Eye Res 1998 66 207 15 9533846 10.1006/exer.1997.0413

[39] Rogers BS Symons RC Komeima K Shen J Xiao W Swaim ME Gong YY Kachi S Campochiaro PA Differential sensitivity of cones to iron-mediated oxidative damage. Invest Ophthalmol Vis Sci 2007 48 438 45 17197565 10.1167/iovs.06-0528

[40] Gomme PT McCann KB Transferrin:structure fonction and potential therapeutic actions. DDT 2005 10 267 73 15708745 10.1016/S1359-6446(04)03333-1

[41] Lukinova N Iacovelli J Dentchev T. Wolkow N Hunter A. Amado D Ying GS Sparrow JR Dunaief JL Iron chelation protects the retinal pigment epithelial cell line ARPE-19 against cell death triggered by diverse stimuli. Invest Ophthalmol Vis Sci 2009 50 1440 7 19182262 10.1167/iovs.08-2545 PMC2665187

[42] Jeanny JC Picard E Jonet L Vesvres M-H Sergeant C Guillou F Behar-Cohen F Courtois Y Human transferrin protects photoreceptor degeneration in 2 animal models: RD 10 mice and RCS rats.19^th^ biennal meeting of the International Society for Eye Research2010p. 256

[43] Age-Related Eye Disease Study Research Group Risk factors associated with age-related macular degeneration. A case-control study in the age-related eye disease study: Age-Related eye disease Study Report Number 3. Ophthalmology 2000 107 2224 32 11097601 10.1016/s0161-6420(00)00409-7 PMC1470467

